# Development, Therapeutic Evaluation and Theranostic Applications of Cubosomes on Cancers: An Updated Review

**DOI:** 10.3390/pharmaceutics14030600

**Published:** 2022-03-09

**Authors:** Yosif Almoshari

**Affiliations:** Department of Pharmaceutics, College of Pharmacy, Jazan University, Jazan 45142, Saudi Arabia; yalmoshari@jazanu.edu.sa

**Keywords:** cubosomes, cancer, theranostic, nanoparticles, lipid, self-assembly

## Abstract

Cancer is a group of disorders characterized by aberrant gene function and alterations in gene expression patterns. In 2020, it was anticipated that 19 million new cancer cases would be diagnosed globally, with around 10 million cancer deaths. Late diagnosis and interventions are the leading causes of cancer-related mortality. In addition, the absence of comprehensive cancer therapy adds to the burden. Many lyotropic non-lamellar liquid-crystalline-nanoparticle-mediated formulations have been developed in the last few decades, with promising results in drug delivery, therapeutics, and diagnostics. Cubosomes are nano-structured liquid-crystalline particles made of specific amphiphilic lipids in particular proportions. Their ability to encapsulate lipophilic, hydrophilic, and amphiphilic molecules within their structure makes them one of a kind. They are biocompatible, versatile drug carriers that can deliver medications through various routes of administration. Many preclinical studies on the use of cubosomes in cancer treatment and theranostic applications have been conducted. However, before cubosomes may be employed in clinical practice, significant technical advances must be accomplished. This review summarizes the development of cubosomes and their multifunctional role in cancer treatment based on the most recent reports.

## 1. Introduction

**Figure 1:**
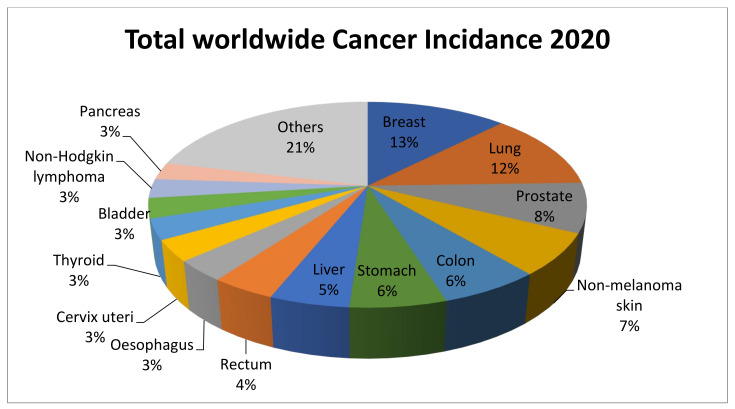
Worldwide distribution of the estimated new cases of cancer in 2020. The data were extracted from earlier published data [5].

Cancer is a spectrum of diseases characterized by abnormal gene functioning and altered gene expression patterns. They arise due to the body’s uncontrolled proliferation of cells and cells invasion to neighboring tissues. In 2020, it was estimated that there would be around 19 million newly diagnosed cases of cancer and approximately 10 million cancer deaths worldwide [1]. Health concerns aside, cancer is also viewed as an economic issue, including in advanced economies. In the United States, it is anticipated that overall Medicare healthcare spending will increase from 3.6 trillion in 2018 to 6 trillion in 2027 [2]. The statistical representation of incidence of cancer is highlighted in Figure 1. Many treatments are available for cancers, such as surgical removal of the tumor or therapies such as chemotherapy, immunotherapy, radiotherapy, hormone therapy, targeted therapies, and stem cell transplant. As the accessible options for cancer patient treatment, each modality has distinct and general advantages and drawbacks [3]. These pros and cons are constantly determined individually, which is why treatment regimens are frequently established utilizing a combination of therapy types to give cancer patients a larger basket of appropriate treatments [4].

There is a high incidence of solid-tumor-related cancer in humans, which necessitates obtrusive cancer treatment methods, such as chemotherapy and surgery to remove tumors if they can be removed, followed by chemotherapy and radiation to destroy the tumor cells that remain [6]. Chemotherapy has played a pivotal role as an essential element in solid cancer treatments, and has grown considerably in recent decades due to the shown advantages of adjuvant chemotherapy. Patients with strong performance status have a greater chance of survival and a higher quality of life after receiving chemotherapy for solid tumors. In contrast, patients with poor performance status are more likely to have a greater chance of symptomatic improvement following chemotherapy [7,8]. Methotrexate was the first-ever introduced chemotherapeutic drug in 1956; since then, chemotherapy has been effectively used and has treated many cancer patients throughout the years. Even though it is an integral part of cancer treatment, chemotherapy faces many challenges [9]. Firstly, it has a unique effect on each individual and might not even work as effectively for some. Secondly, the severe side effects might harm the patient and their quality of life, rather than curing them effectively. Lastly, as a result of their scarcity and high cost, many medications are out of reach for most people [10]. Numerous strategies are available to enhance chemotherapy’s activity and minimize its harmful effects, including discovering new drugs. Nonetheless, as per the latest study, developing a new medication approved for commercialization is expected to cost USD 2.6 billion [11]. Chemotherapeutic medicines can be rendered successfully with negligible side effects if innovative drug delivery methods are used, decreasing loopholes such as side effects and costs by providing accuracy, targeted administration, and low-dosage loading.

Selective targeting of the disease site within the body is one of the significant challenges and needs for drug delivery [12,13]. In general, chemical agents are distributed evenly in the body, thereby enabling the drugs to distribute to every part of the organ system. In contrast, chemotherapeutic agents are supposed to target only the tumor site, or the majority of the drugs are considered to reach the target site. This is because of the unnecessary side effects the chemotherapeutic drugs could bring to those healthy areas of the body. Hence, a targeted drug delivery system must meet two functional responsibilities, as it has two components present in the delivery system [14]. The primary function of the carrier is to identify the target, thereby enabling the drugs within it to provide therapeutic efficacy [15]. Such drug delivery systems are widely and successfully delivered using unique particles that come under the category of nanoparticles. Nanoparticles are widely characterized as materials with a particle size between 1–100 nm [16]. As a result of the extensive use of varied nanophases and nanostructures, nanoparticles have come to play an increasingly essential role in a wide variety of scientific fields such as drug delivery, diagnostics, bioengineering, and sensors, amongst other functions [17]. They excel in their role as a connection between the physical sciences and the biological sciences, which is especially significant in the development of pharmaceuticals and the practice of biomedicine.

Successful therapy is dependent on the bioavailability and steady-state in the blood level of the nanoparticle, and the surface properties of the nanoparticle play an essential role in this. For instance, nanoparticles are frequently modified to become more hydrophilic, allowing them to remain in the bloodstream for more extended periods, resulting in a larger concentration of drugs at the tumor site and a longer half-life overall [18]. When making nanoparticles more hydrophilic, one of the most commonly used hydrophilic compounds is polyethylene glycol (PEG). Furthermore, one of the advantages of these surface modifications is the capacity of such nanoparticles to elude the attack of opsonic proteins in the bloodstream, preventing nanoparticle opsonization and phagocytosis [19,20]. Surface-treated nanoparticles have lower toxicity and greater therapeutic potential at low doses. For example, the frequency of cardiotoxicity caused by doxorubicin is considerably lower in doxorubicin-loaded PEGylated liposomes than in doxorubicin alone [21].

The pathophysiology, size, and other specific tumor features are considered when selecting the appropriate drug delivery materials from nanoparticles. Precise form, size, and surface features of the nanoparticles utilized as drug delivery carriers in tumors are required, because physical attributes play a critical role in therapeutic efficacy [22]. Compared to bigger carriers, these features and their nanoscale size allow the carrier to travel more freely throughout the human body. In cancer treatment, for instance, particles smaller than 10 nm are not generally recommended since they have a greater likelihood of leaking into the vasculature and causing increased renal filtration, in addition to normal cell toxicity [23]. Larger-sized particles remain in the circulatory system for extended and anticipated periods and allow the carrier-associated therapeutic agents to be released into the site of action without causing significant systemic fluctuations or adverse health effects. Therefore, particle sizes more than 10 nm and less than 100 nm are preferable in cancer [24].

Numerous nanoparticles have been produced and are now being utilized to exhibit all standard physical properties. They are categorized as organic, inorganic, or hybrid. Organic nanoparticles such as lipoproteins, polymeric nanoparticles, and polymeric micelles are widely employed [25]. Liposome-based nanoparticles have been extensively employed in treating breast and prostate cancer. They are unique in their capacity to change the lipid bilayer of cancer cells without causing damage to the surrounding healthy cells, so are well tolerated in formulations. Due to the improved biodegradability and compatibility of polymer-based nanoparticles such as polylactic-co-glycolic acid, they are commonly used as drug carriers. At the same time, polymeric micelles benefit from increasing the absorption of insoluble anticancer agents and delivering them efficiently to the target [26]. Various investigations have been conducted on inorganic nanoparticles, including gold, carbon nanotubes, magnetic nanoparticles, and silica nanoparticles. They have several benefits over organic materials, including increased drug accumulation in tumors, provided by gold and the encapsulation of silica’s most significant quantity of anticancer medications [27]. Finally, hybrid nanoparticles such as liposome-silica hybrids, chitosan-carbon hybrid nanotubes, cell-membrane-coated nanoparticles, and lipid–polymer hybrid nanoparticles have demonstrated considerable anticancer activity [28].

**Figure 2:**
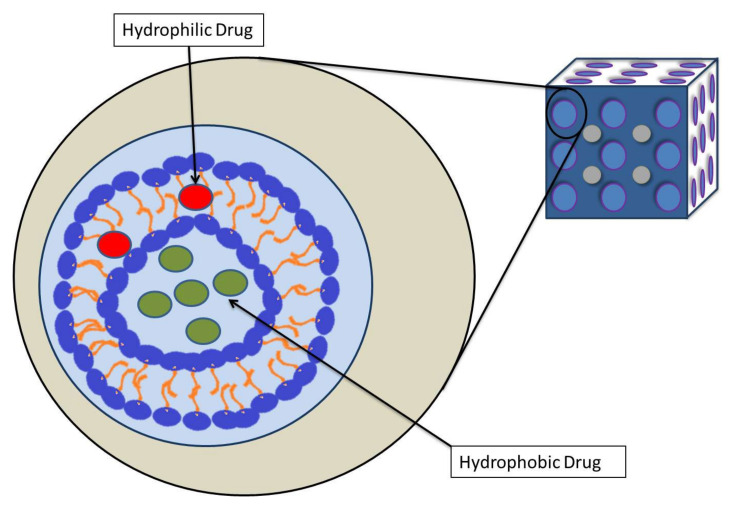
Structure of cubosomes.

Cubosomes are a new type of nanoparticle that contain a lipid cubic phase and are stabilized by a polymer-based outer corona (Figure 2). They are among the latest nanomaterials to gain traction in pharmaceutical drug development and delivery. The development of cubosomes has mainly depended on amphiphilic lipids [29]. They have several benefits over other nanomaterials, including the ability to overcome some of the primary disadvantages of cubic phases, such as excessive viscosity. Many studies have been conducted to assess the potential use of cubosomes in various disease models, including hepatoprotection [30], skin infections [31], ophthalmic applications [32], Alzheimer’s disease [33], ENT infections [34], and so on. Cubosomes have numerous physical qualities that can be employed in cancer medication delivery, where the major problems include toxicity, drug delivery, cost-effectiveness, etc. In the last decade, the use of cubosomes in the administration of anticancer drugs has increased. However, there is yet to be a thorough review that includes all of them, focusing on their relationship to cubosome physical qualities and physiological functions. As a result, the present literature review will concentrate on the physical properties of cubosomes and their development, and a complete report on anticancer research conducted on cubosomes will be discussed based on the recent literature published in the field.

## 2. Cubosomes

**Figure 3:**
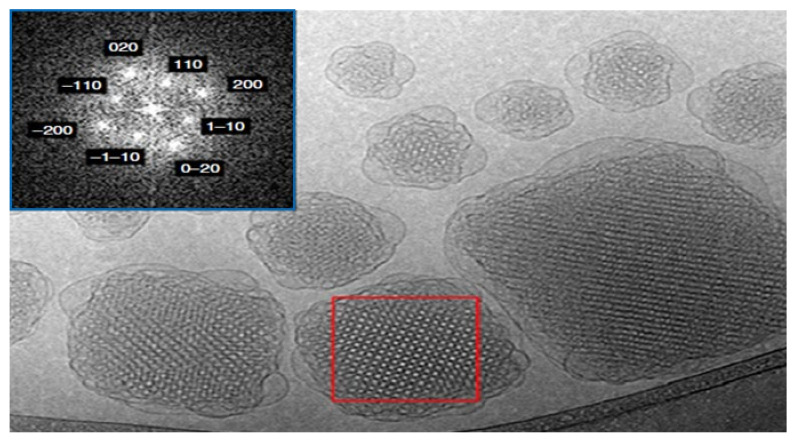
An image of a cubosome taken using cryogenic transmission electron microscopy (cry-TEM). The red box denotes the well-ordered structure created in the particles within and near the water matrix contact with a vesicular structure. The fast Fourier transform (FFT) of the red box region is shown in the photo inset in a blue-colored box and is utilized to determine the structure of the liquid-crystalline particles, which was also validated by SAXS analysis. Scale bar, 100 nm. Source: Reproduced from [41].

Cubosomes are a novel colloidal dispersion with a bicontinuous cubic phase in water, which surfactants have stabilized to produce a unique, nanoscale, structured system. They are typically between 10 and 300 nm in size and are primarily employed to transport various chemical compounds in living and non-living matter [35,36]. In addition to their other nanoparticle properties, these cubosomes are unique in their ability to encapsulate lipophilic, hydrophilic, and amphiphilic molecules within their cubosome structure [37]. Figure 3 shows a cryogenic transmission electron microscopy (cryo-TEM) picture of a cubosome. Cubosomes, are more stable than liposomes and have a far greater potential to encapsulate hydrophobic molecules because of their liquid-crystalline membrane structure [38]. In addition to chemical compounds, cubosomes may also be utilized to transfer various proteins into biological systems, known as proteocubosomes. Peptides and nucleic acids can be delivered with expected loading and release [39]. Among cobosomes’ many benefits is the capacity to transport several proteins through the water channel of proteocubosomes, ensuring the stability and delivery of molecules to their intended biological targets without degradation by enzymes [40].

There are several benefits to using cubosomes over conventional cubic–phase drug delivery methods. This product has a long shelf life due to its high level of bio-adhesives, superior dermal penetration, ease of formulation, higher drug loading capacity, and greater stability at any dilution level, as well as its higher resistance to breakage and protection of enzyme attack-liable drugs within the cubic phase. It is economical, cost-effective, biologically compatible, and non-hazardous. Compared to other prominent categories of nanoparticles, cubosomes have various advantages. For instance, compared to liposomes in contact with cellular surfaces, the main benefit of cubosomes over liposomes is their liquid-crystalline arrangement, which may offer continuous drug release over lengthy periods [42]. Additionally, cubosomes possess a greater volume to accommodate increased quantities for drugs, resulting in better payload, less viscosity and a less hydrophobic core than liposomes [43]. Cubosomes have a significant advantage compared to dendrimers. Dendrimers have potential toxicity issues related to charges and the nature of the building blocks, while cubosomes use biodegradable, biocompatible and bio-adhesive lipids. However, the formation of increased viscosity during large-scale manufacture and a few issues with retention of hydrophilic drugs remain its principal drawbacks [44]. Even though there are some disadvantages, due to unique benefits among other nanomaterials, self-assembly capacity, better encapsulation of drugs, biological transportation, and applications in diagnosis, they have been used in a variety of applications for over two decades [45]. When a lipid bilayer is applied to a twisted three-dimensional surface with minimal surface formation comprising water and lipid phases, cubosome development occurs under exactly regulated temperature conditions. Cubosome formation may be divided into three types: primitive (P-surface), double diamond (D-surface), and gyroid (G-surface), all of which are in a favorable structural variation so that drug delivery to different biological targets is possible. Although these cubosomes have a microstructure comparable to their parent cubic phases, they are distinguished by their decreased viscosity as dispersions and their acquisition of a high surface area as equating [44]. This nanodispersion appears to be a potential approach for overcoming the major disadvantages of cubic phases.

## 3. Development of Cubosomes

### 3.1. Self-Assembly of Amphiphilic Liquids

Self-assembly is a process in which disordered molecules come close together and spontaneously create a structurally orderly arrangement through reciprocal interaction [46]. Amphiphilic chemicals have both hydrophilic and lipophilic components; long-chain hydrocarbon chains make up the lipophilic end of amphiphiles, while the hydrophilic end might be ionic or non-ionic. It has been demonstrated that amphiphilic surfactants, which include both hydrophilic and hydrophobic components, may self-assemble to form highly organized aggregates in the presence of aqueous fluids. It is possible to find such formed assemblies in the form of open or closed lipid bilayer structures, as well as micelles or, in some cases, inverted micelles [47].

The theory behind the self-assembly of amphiphilic liquids is associated with two principles: opposing force and packing parameter [48]. According to the principle of opposing forces, molecular arrangements of amphiphilic molecules in a polar solvent reduce free energy. The solvent may pass through them and expose the hydrophilic regions to the aqueous environment, while protecting the hydrophobic portions from the solvent. At this point, opposing forces begin to arise, as hydrophobic contacts occur at the interface between hydrophobic hydrocarbon tails and the hydrophilic head groups on the amphiphilic molecules. The hydrophobic effect phenomena emerges due to this approach [49]. The packing parameter principle describes the lipid aggregates that form preferentially with any lipids. Israelachvili [50] was the first to suggest this idea; it was modified by Abdelkader [48] later, which he did using the formula shown below.

p=v/al

At the amphiphilic interphase, this formula describes the shape of the aggregates generated and their curvature. The packing parameter is ‘*p*’, the volume of the hydrophobic chain is ‘*v*’, the optimal surface area of the polar head is ‘*a*’, and the length of the hydrophobic chain is ‘*l*’.

### 3.2. Amphiphilic Lipids for Cubosomes

Cubosomes are made up of three components that self-assemble to produce the lipid bicontinuous cubic phase: amphiphilic lipids, stabilizers, and drug molecules. However, the basic skeleton formed of cubosomes is determined by the properties of the amphiphilic lipids used. To produce cubosomes, two frequently used and distinct types of amphiphilic lipids are employed: monoolein and phytantriol (PHYT), the latter of which is also known as glycerol monooleate (GMO) [51]. GMOs are made up mostly of monooleate and glycerides of oleic acid and other fatty acids. They have a Pn3m cubic-phase structure and pass through inverted micellar and lamellar phases when exposed to excess water, and temperatures ranging from room temperature to 80 °C [52]. Physically, they are colorless, polar unsaturated monoglycerides with a melting point of 27–35 °C. Because they are amphiphilic, they nurture in both lipophilic and hydrophilic ways. Due to the presence of hydrocarbon chains in the tail region and hydroxyl groups in the head region, the amphiphilicity of this compound may be explained by its ability to establish hydrogen bonds with water in aqueous media [53]. Since GMO is a biodegradable substance, it is also employed in the food industry, mostly as an emulsifier [54].

Phytantriol (PHYT), on the other hand, is remarkably similar to monoolein. Chemically it is 3,7,11,15-tetramethyl-1,2,3-hexadecanetriol (C_20_H_42_O_3_) [55]. It is amphiphilic and biocompatible, and it shows extremely comparable phase-change tendencies to monoolein with excess water content at higher temperatures. It is often employed in cosmetic preparations and has higher structural stability owing to the presence of a saturated phytanyl backbone and the absence of ester linkage, while monoolein is sensitive to esterase-catalyzed hydrolysis [54]. The formation of Q_II_ and H_II_ stable phases is essential for cubosomes. It is worth noting that the production of Q_II_ and H_II_ stable phases in the phytantriol/water system in PHYT occurs at a lower temperature (40 °C) than monoolein (80 °C) [55]. They have a high skin penetration capability, exceptional purity, and moisturizing ability. Furthermore, when synthesized in a PHYT-based liquid-crystalline matrix, sustained release dosage forms of hydrophilic pharmaceuticals demonstrated improved release capability [55,56]. As a result, in cubosome preparations, PHYT is usually seen to be a superior alternative for monoolein.

### 3.3. Stabilizers

The inclusion of stabilizers is a crucial component of the cubosome-making process. They work by forming a protective layer over the cubosome structure, preventing aggregation and increasing dispersion stability by preventing amalgamation with the bulk cubic phase [57]. In addition to assisting in preparing long-term stable cubosomes, the stabilizers also aid in the cellular uptake of the drugs contained within them by increasing the cellular membrane permeability. This reduces the need for toxic doses of drugs while also targeting cells of interest using compound-loaded cubosomes, which is particularly important. It is evident that although the fundamental role is to control the phase morphology of lipid mixtures, a large proportion of the stabilizers remains on the surface of the cubosomes, with just a tiny amount intercalating into the phospholipid bilayers of the cells [58]. The number of stabilizers that may intercalate into the lipid membrane varies depending on the kind and quantity of the stabilizers.

Block copolymers are the most often-utilized stabilizers in the manufacture of cubosomes, accounting for more than half of all applications. F127 (Poloxamer 407), a triblock copolymer, has long been considered the gold standard for non-lamellar lyotropic liquid crystal (LLC) lipid nanoparticles. Approximately 12.6 kDa in molecular weight is composed of polyethylene oxide and polypropylene oxide (PEO-PPO-PEO) [59]. They have a hydrophilic–lipophilic balance of ≥23 to enable effective dispersion and can produce many months-long stable cubosomes, with their PPO (hydrophobic domain) parts found on the surface of the cubosome or inside the lipid bilayer and the PEO (hydrophilic domain) parts exposed to adjacent aqueous phase [59,60]. In most cases, F127 is used at a concentration of 20% *w*/*w*. Studies have shown that while increasing the concentration of F127 results in smaller particles in the dispersion, doing so also encourages the formation of vesicular particles rather than the expected nano-structured particles of the cubic matrix. In any case, decreasing the quantity of F127 to shallow levels is difficult, since enough F127 is required to form the p-type cubic phase, which is responsible for establishing a stable colloidal dispersion [61]. There are very few studies on the safety and biocompatibility of F127, which makes it hard to anticipate its protective nature and other related covert functions at the cellular level without more research. However, since it is a steric stabilizer, we may be confident in its high degree of safety; this is because steric stabilizers are generally regarded inert at the cellular level, which is supported by the literature [61]. To ensure that cubosomes remain within their safe operating window, it is strongly advised that non-Pluronic polymers be used when the circumstances allow it.

Various stabilizers may be utilized in the production of cubosomes in addition to F127. Propylene glycol (PG), polyethylene glycol 400 (PEG400), polysorbate 80 (Tween 80), and 2-methyl-2,4-pentanediol (MPD) are some of the alternatives to F127 that are often employed with PHYT-based cubosomes, although they are not the only ones [62]. They outperform F127 in terms of their ability to include physiologically active diglycerides, to have a specified molecular weight, and their preference for target drug delivery. PHYT has higher hydrophobicity than GMO, which makes it less flexible. As a result, distinct phases are created because the different stabilizers are used as alternatives. For example, propylene and polyethylene glycol, when combined with PHYT, generate cubic, lamellar, and non-ordered liquid phases, but the addition of MPD to PHYT results in the creation of a sponge phase [63].

As an alternative to Pluronics, numerous different efforts have been undertaken to investigate copolymers that are alternative replacements for Pluronics in order to solve the difficulties associated with precision synthesis, which is required in Pluronics synthesis. For instance, Cho and colleagues [64] showed that binary blends of block copolymers may self-assemble into the required nanostructure in solution by varying their composition within the blend. They were able to accomplish this by manipulating the structural parameters of a binary block copolymer blend through composition control. Two block copolymers that share the repeating units in both polymer blocks co-assemble into the desired structures, which range from spherical micelles to inverse cubic and hexagonal mesophases, among other things. This can be achieved without relying on the precise synthesis of a correspondingly designed block copolymer. LDBCs are amphiphilic linear-dendritic block copolymers composed of hydrophilic dendritic poly (ether-ester) (PEE) blocks based on 2,2-bis(hydroxymethyl)propionic acid and hydrophobic linear poly(styrene) (PSt) blocks. Liu and colleagues [65] demonstrated the self-assembly of a series of amphiphilic linear-dendritic block copolymers (LDBCs). This is accomplished by changing the common solvent from tetrahydrofuran to dimethylformamide, which results in an increase in the formation of wormlike micelles and/or spheres during the self-assembly process.

### 3.4. Preparation of Cubosomes

The formation of cubosome dispersions with nanometer-scale structures comparable to a bulk cubic phase is the major goal in the formulation of cubosomes; nevertheless, the dispersion must have a viscosity equivalent to water to achieve this goal successfully [29]. Cubosome preparation is relatively more common in the pharmaceutical industry than the preparation of the corresponding reverse non-lamellar phases, owing to the ease with which cubosomes can be prepared and their greater ability to deliver a broader range of pharmaceuticals, particularly those that are injectable [66]. Since 1983, various approaches have been explored to attain these features, therapeutic delivery, and the production of cubosomes using nanostructured aqueous suspensions. However, the top-down and bottom-up methodologies are the two most important methodologies now in use. Using a common colloidal stabilizer known as P407, both of these approaches prevented the development of aggregation and the production of cubosome dispersion [54].

The top-down strategy is the first method used to create cubosomes [67]. By adopting a two-step method, this approach begins with acceptable starting materials, then carves the usefulness out of them. It is necessary to produce a bulk cubic viscous phase; the viscous bulk cubic phase is made by mixing two components: lipids and stabilizers. Aqueous medium is next added to the mixture produced in the first stage, along with applying high-energy shear forces such as sonication or homogenization until the sample becomes homogeneous. At that point, cubosomes develop due to the first step. Maintaining the optimal temperature throughout this phase is critical, since failure to do so may result in poor-quality cubosomes [45]. Therefore, the generated cubosomes will be stable against the aggregation of cubic phases for at least 12 months. Cubosomes developed in this manner produce a transparent, stiff gel composed of cross-linked polymer chains that have been expanded by water. They may be found in vesicles, such as lamellar liquid-crystalline phase-dispersed nanoparticles or vesicle-like structures [54]. Top-down sonication techniques have the major benefit of producing repeatable and stable cubosomes without additional solvents. A re-examination of phase behavior is not necessary, and the risk of toxicity to cells is minor or non-existent. However, this technology has certain drawbacks, such as the need for greater energy input to disperse the cubic phase into nanocubosomes, due to the creation of the viscose cubic structure as a precondition [44].

The second approach for synthesizing cubosomes is the bottom-up method, also referred to as the solvent dilution method or the liquid precursor method [68]. In this method, cubosome precursors may be transformed into the crystallized form on the molecular length scale while remaining at room temperature. Spicer et al. first described this process, wherein they made nano-structured building blocks and converted them into finished materials [69]. They advocated for the dispersion of combinations of monoolein–water–ethanol phase and cubosomes created by a dilution (nucleation) process to achieve the desired results. Cubosomes are formed by applying minimal shear stresses in excess of the water phase, are stable, and have unique structures [47]. The cubosomes formed exhibited less polydispersity and less vesicle formation than the cubosomes made using the top-down sonication approach. Furthermore, the bottom-up method has several advantages over the top-down method, including less energy due to avoiding strenuous fragmentation, the inclusion of thermolabile materials, and the generation of small particle cubosomes due to a unique technique. The uniform dispersion of stabilizers used in this method leads to the development of long-term stable cubosomes and the ability to scale up to industrial batches [54].

### 3.5. Characterization of Cubosomes

The physical parameters of cubosomes may be determined in various ways. Firstly, photon correlation spectroscopy, also known as quasi-elastic light scattering, is a technique that is often used to gather information on the aggregated species present in a lipid layer size distribution. Photon correlation spectroscopy may be used to study the interaction between light and matter using the theory of diffusion coefficient of cubosome particles in Brownian motion. A suitable solvent is used to dilute the cubosome samples, after which they are subjected to light scattering at an intensity of 300 Hz, and measurements are carried out at 25 °C. Due to the presence of a solute particle, the light scatters as a function of time, reflecting the information contained inside cubosomes. Data is gathered and evaluated in size, shape, and flexibility [70,71]. Secondly, polarized light microscopy may be utilized to investigate the vesicular optical birefringence of the refractive index caused by the surface coating of cubosomes materials. This approach has previously been claimed to be used to detect the existence of crystals in colloidal systems, where it divides cross-polarized light [72].

Apart from these two techniques for characterizing cubosomes, three other strategies are critical in cubosomes. They are small-angle X-ray scattering (SAXS), cryo-TEM (cryo TEM), and Energy-dispersive X-ray analysis (EDAX) [73,74]. SAXS is a fairly simple process that is conceptually similar to light scattering. In the case of SAXS, high-energy X-rays are generated from electrons and are directed towards cubosome samples in a practically wavelength-independent manner. The diffracted patterns are translated to plots of intensity against a q value, which provides the ring’s unique properties. They denote the unique arrangement of several groups inside a cubosome sample [70,75]. Apart from SAXS, additional new techniques for cubosome characterization include cryo transmission electron microscopy (cryo-TEM). This is a powerful tool for characterizing the morphology of soft matter dispersions, polymeric nanoparticles, and biological materials [76]. This approach is based on the ultra-rapid conversion of a thin fluid suspension film to a vitrified low–vapor–pressure specimen suitable for electron microscopy. Diffraction spots may be created and utilized for phase by obtaining fast Fourier transforms of the lipid structures within the pictures [77]. The use of cryo-TEM offers the potential benefit of avoiding fixation and, as a result, avoiding the possibility of lipid breakdown and artifact development. Furthermore, the high-resolution images acquired will aid in the study of materials with a variety of structures, as well as diverse morphologies and sizes, among other things. Despite these benefits, the primary drawbacks of cryo-TEM are the difficulty in sample preparation and the difficulty in aligning pictures once they have been processed. According to the literature, cryo-TEM is particularly beneficial when used in conjunction with other methods such as SAXS and photon correlation microscopy [78]. Energy-dispersive X-ray analysis (EDAX) is a technique used to measure nanoparticles by SEM. In this technique, the cubosomes are analyzed by activation using an EDS X-ray spectrophotometer, which is generally present in modern SEM. The basic principle of EDAX is the generation of X-rays from a specimen through the electron beam. The X-rays are generated according to the characteristics and nature of the elements present in the sample [74]. Various studies have been carried out to characterize the cubosome nanoparticles using EDAX [79,80].

## 4. Physiological Properites and Drug Delivery of Cubosome

The major advantage of cubosomes as nanoparticles is that they can accommodate hydrophilic, hydrophobic, and amphiphilic drug molecules. Cubosomes, according to the literature, have a number of other properties that make them attractive for use as drug-delivery vehicles. For example, they demonstrate biocompatibility; bio-adhesion; the protection of drug molecules against oxidation, hydrolysis, and deamidation processes; and the protection of protein molecules against denaturation, precipitation, aggregation, and surface adsorption. Additionally, they have been shown to be an effective delivery method over an extended period. These issues continue to be a barrier to achieving an optimal treatment response and patient compliance in the therapeutic region. Cubosomes are on the right track in this regard and have been suggested for use in a variety of chemical, peptide, and protein delivery systems.

Cubosomes’ physical properties make them ideal for oral drug delivery. Precipitation of oral medications is highly protected by cubosomes owing to the cubosomes’ lyotropic structure and consequent trapping of water-soluble compounds in the lipid bilayer absorption membrane. Another benefit of cubosomes in oral delivery is their potential to improve molecular absorption thanks to their bio-adhesive properties and surfactant production in the gastrointestinal system [81,82]. According to a study conducted by Mohsen et al., cubosomes have been demonstrated to increase the bioavailability of Coenzyme Q10, an antioxidant used in the treatment of liver disorders, by forming highly bioavailable and regulated drug formulations [83]. Cubosome formulation, developed by Chung et al. previously, has successfully enhanced oral insulin absorption [84].

The most significant problem with topical pharmaceutical delivery is enabling the medication to penetrate the skin. Several dosage forms have been produced to address this problem, and any penetration enhancers that have been developed have also been studied. While this is true, a critical difficulty still exists, which is the increase in the thermodynamic activity of active molecules without increasing their concentration in the skin, for the skin barrier to be more readily penetrated. Many studies have been successfully carried out to resolve these problems. Morsi and colleagues created cubosome formulations including silver sulfadiazine, monoolein, and the F127 stabilizer, which have been shown to be beneficial in treating deep second-degree burns [85]. Furthermore, it has been demonstrated that colchicine manufactured as a cubosome transdermal preparation improves topical medication absorption, compared to when the drug is administered orally [86].

Cubosomes have also shown considerable benefits in the delivery of drugs through intravenous and intranasal routes. Cubosomes may aid in the transfer of colloidal substances without obstructing capillaries. Additionally, they may minimize drug plasma–protein interactions, increasing drug molecules’ bioavailability and stability. Intranasal cubosomes can deliver drugs directly to the central nervous system (CNS) by crossing through the blood–brain barrier [87]. Thus, cubosomes are a non-invasive medication delivery system for centrally acting drugs in various disorders. Cubosomes are beneficial in both routes of delivery. For example, Elsenosy and his team found that Duloxetine can be quickly delivered to the brain by in situ cubosome gel, which has better pharmacological effects [88].

Cubosomes have been tested in many ways to exert their delivery capacity as nanoparticles with several disease models. However, very few studies have been conducted to evaluate the toxicity profiles of ingredients and stabilizers. Most studies of the sort have been tested with the help of in vitro analysis using MTT assays. Most of these studies evaluated the toxicity of phytantriol and monoolein-based cubosomes and stabilizers, such as Pluronics F108 and F127, and PEG conjugated lipids. The toxicity profiles for these materials were found to be very specific among the cell line chosen. For instance, F127 surfactants were found to be non-cytotoxic up to a concentration of 25 µg/mL in A549 and CHO cells, but were found to be highly toxic in HEK and L929 cells [89,90]. At the same time, F108 was found to be nontoxicnontoxic in Hela and HEK 293 cells up to 80 µg/mL concentrations [91]. Hinton et al. compared the effects of F127 and the lipids monoolein and phytantriol on toxicity using an Alamar Blue assay. phytantriol-based cubosomes were found to be more toxic than monoolein-based ones. It was concluded that the cubic phase and its constituent lipid are the primary sources of toxicity, not the Pluronic [92]. Murgia and co-workers found that while F127 itself is nontoxic, they speculate that monoolein promotes the internalization of F127 by decreasing its hydrophilicity and that, once internalized, its amphiphilic nature allows it to exert toxic activity towards the mitochondrial and other nuclear membranes [93]. Studies carried out so far undoubtedly showed that the cubosome formulation’s toxicity may be due to the ingredients used in the formulation, or sometimes due to the drug or protein loaded in it. Hence formulation optimization and toxicity studies would need to be performed for individual cases.

## 5. Anticancer Activity of Cubosome

In cancer, the main challenges faced during the treatments are the targeted delivery of drugs to reduce the side effects and drug resistance by overcoming drug efflux transporters. Cubosomes are highly significantly taken into account in experiments in the cancer drug delivery area. They have achieved both targeted drug delivery and reduction in drug resistance. Moreover, as part of cancer drug treatment, cubosomes have also been used in immunotherapy. Studies have shown that cubosomes have improved the pharmacokinetics and safety profiles of the loaded drugs (Table 1).

### 5.1. Cubosomes in Colorectal Cancer

Colorectal cancer is one of the most common and diagnosed solid malignancies globally. It appears in the colon or rectum mucosa as a malignant tumor [111]. Based on the development, they have divided the process into five stages. Patients’ survival rates declined as the stages progressed, but the patient might be treated with surgery and chemotherapy if the diagnosis was made sooner. Despite this, medication resistance and adverse effects continue to be critical obstacles. Nanoparticle technology has been extensively employed for effective medication release, cancer cell targeting, and reducing chemotherapy side effects [112].

**Figure 4:**
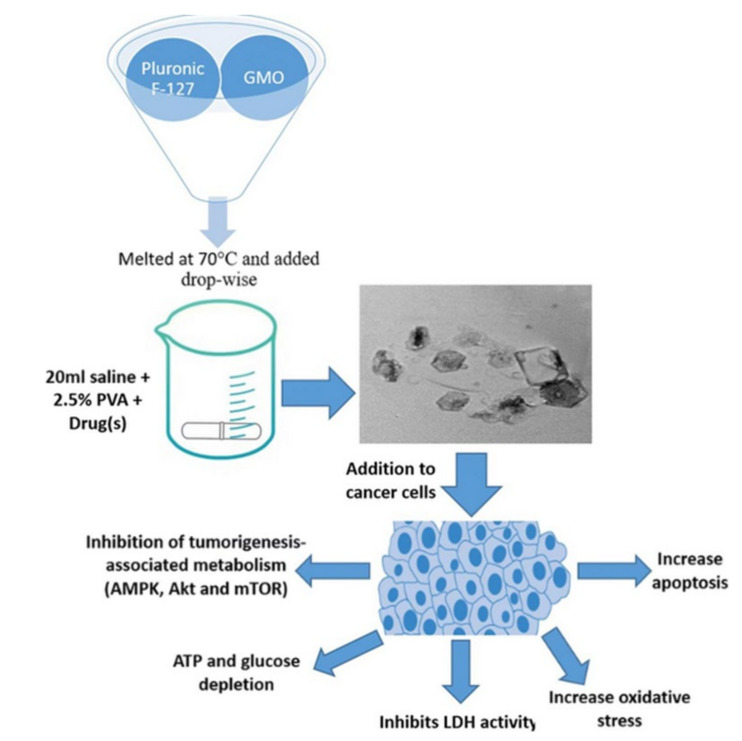
The emulsification process is used to manufacture cisplatin and cisplatin–metformin nanocubosomes. When CRC cells are treated with drug-loaded nanocubosomes, multiple metabolic pathways, including the AMPK/mTOR and Akt/mTOR pathways, are significantly inhibited. As a consequence of the depletion of ATP and glucose, there is a rise in oxidative stress and apoptosis. Another way the nano-cubosomes cause cytotoxicity is by inhibiting LDH activity, which leads to caspase-3 activation. Source: Reproduced from [94].

The application of cubosomes nanoparticles has experimented with several times in colorectal cancer [113,114]. Saber and colleagues had explored reducing the toxicity of cisplatin, a major drug used in colorectal chemotherapy. They demonstrated significant anticancer efficacy in vitro against human colorectal cancer cells compared to unformulated cisplatin. In their study, nano cubosomes have prepared with GMO and Pluronic F127. According to the study, metformin’s cytotoxicity is increased when combined with nano-cubosome cisplatin. The IC_50_ in colorectal HCT-116 cells after treatment with cisplatin was 15 µM, whereas the IC_50_ of cisplatin-loaded nano-cubosomes was 9.6 µM. Additionally, the determination of cisplatin concentrations at the intracellular level 48 h after treatment with the same concentration of (7 µM) cisplatin, cisplatin nano-cubosomes, and cisplatin-metformin nano-cubosomes revealed a significantly increased uptake of the drug due to nano-cubosomes drug incorporation. This was shown clearly in colon cells treated with nano-cubosomes, where a 1.6 fold increase in cisplatin concentration was found compared to untreated cells. Moreover, cubosome nanoparticles induced death in CRC cells by disrupting numerous metabolic pathways (e.g., mTOR inhibition, AMPK activation), lowering glucose levels, and decreasing energy levels (Figure 4) [94].

Magdy et al. examined metformin alone in colorectal cancer. This anti-diabetic medication has already shown its ability to decrease the incidence of colorectal adenoma and thereby improve patient life. Magdy and colleagues developed a monoolein, and water-based metformin cubosome dispersion stabilized with Pluronic F127. The findings indicated that metformin-loaded cubosomes generated much more toxicity in vitro in HCT-116 and Caco-2 colorectal cancer cells than unloaded cubosomes or metformin alone. The cubosomes formulation significantly lowered the formulation’s IC_50_ concentration at which viable cells were destroyed. In HCT-116 cells, the IC_50_ decreased to 20 from 55 mM; however, in Caco-2 cells, it dropped to 28 from 50 mM. Their work demonstrates unambiguously the possibility of incorporating a modest amount of metformin into cubosomes to treat colorectal cancer patients [95].

**Figure 5:**
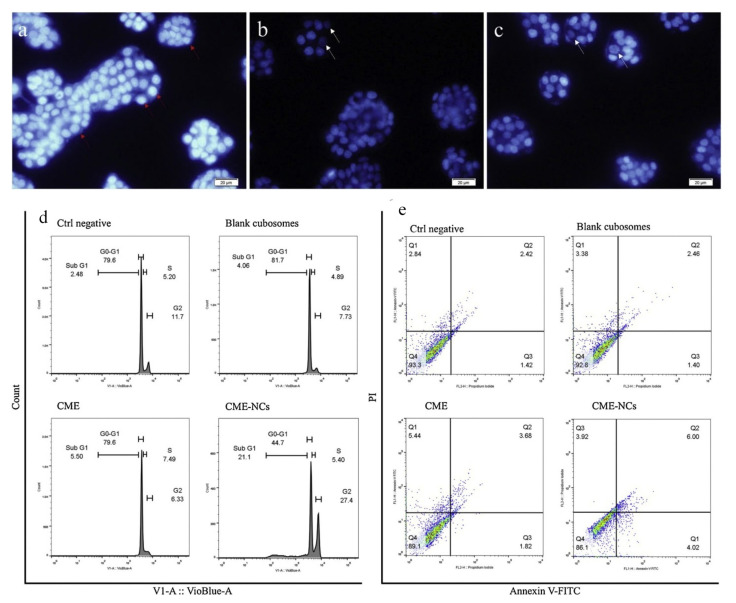
Cornus mas extract-nano carrier induced apoptosis and cell cycle arrest: (**a**) negative control, (**b**) Cornus mas extract treatment; and (**c**) Cornus mas extract treatment with nano carriers presented fluorescence pictures of treated and untreated DAPI stained HT-29 cells (red arrows indicate healthy cell nuclei and white arrows indicate fragmented cell nuclei samples); (**d**) depicts cell cycle analysis, whereas (**e**) depicts cell apoptosis as determined by Annexin V FITC/PI (propidium iodide) labeling. Source: Adapted with permission from [96], Elsevier Masson SAS, 2020.

Aside from using synthetic medications in cubosome formulations, researchers have developed and described a range of natural products that have been integrated into cubosome preparations to study their effects on colon cancer. Anthocyaninsas is a naturally occurring bioactive phenolic constituent in Cornelian cherry (*Cornus mas* L.) fruit. It has significant cytotoxic and antioxidant activity. Its oral bioavailability remains very low due to the destruction of chemicals and participation of gastro microbiota during their metabolism. Aside from that, it was discovered earlier that its increased absorption occurs via the small intestine. Radbeh and colleagues have created enteric-coated nano cubosomes of Cornelian cherry extracts to improve their anticancer activity in colon cancer cells and protect them from gastrointestinal damage. Cubosomes were created by combining glycerolmonooleate with the stabilizer poloxamer^®^ 407 in a proprietary formulation. The findings revealed that the cubosome prepared significantly protects the antioxidant activity of the extract. Its ability to induce apoptosis and cytotoxicity in HT-29 cells increased, with an inhibitory concentration (IC_50_ value) of 1.33 and 1.47 times greater than that of free Cornus mas extract after 24 and 48 h of incubation, respectively. Finally, they discovered that the increase in cell cycle arrest occurs during the G0-G1 phase, contributing to the reported loss in cell viability seen. G0-G1 phase could be a critical factor in the observed reduction in cell viability (Figure 5) [96].

Ginsenosides are a family of natural steroid glycosides and triterpene saponins derived from Panax species. It has been demonstrated to have vast medicinal characteristics, especially in cancer. Ginsenosides is a prodrug that, following intestinal metabolism, transforms into an active product. 20(S)- protopanaxadiol (PPD) is Ginsenosides’ most active and effective anticancer metabolites. Despite being an effective substance for cancer, it is has a downside of weak water solubility and poor penetration of cancer cells. Jin and colleagues have sought to sidestep these pharmacological features by manufacturing cubosome preparations by fragmentation the glyceryl monoolein (GMO)/Poloxamer 407 bulk cubic gels. They investigated its numerous medicinal capabilities in the colon Caco-2 cells model. They have observed that PPD-cubosome formulation may raise the cell permeability apical to the basolateral of PPD at 53 %. This behavior may be attributed to the specific qualities of cubosomes produced, which severed as permeability enhancers and bioadhesive. Hence, the formulation has been proven to be a paradigm for colorectal cancer, notably by the oral route of delivery [97].

### 5.2. Cubosomes in Liver Cancer

Liver cancer is one of the deadliest diseases globally, and in the USA, it is the 4th major reason for cancer-related mortality. It has a poor prognosis rate, and the primary causes for the disease include fatty liver, hepatitis, cirrhosis, obesity, etc. In the first stages, the surgical intervention appears to be beneficial to the patients; however, within the later stages, chemotherapy is adopted. There are limited alternatives to accessible chemotherapeutic medications like sorafenib. The risk of drug resistance to the regimen during the six months is a major concern. [115]. To enhance liver cancer therapy, several novel nanomaterials have been employed presently. Their distinct physical properties might offer targeted drug delivery accuracy and reduced adverse effects. Several efforts have been made to include drugs in cubosomes to boost their therapeutic action.

5-Fluorouracil (5FU) is a potent anticancer drug that has been used to treat solid tumors, particularly liver cancer. However, the substantial adverse effects of 5FU, including hematologic and gastrointestinal complications, preclude its widespread usage in many circumstances [98]. Thus, to reduce the therapeutic dose of 5FU and improve its physical properties, Nasr and colleagues synthesized cubosome dispersions and evaluated their effectiveness in vitro in human hepatoma HepG2 cells and in vivo in rats. They employed a cubic gel phase of monoolein, water, and Poloxamer 407 as a stabilizer. Their formulation demonstrated a quick release of around half of the entrapped 5FU during the first hour and a gradual release of the remainder. The biodistribution of 5FU in the rat liver was substantially greater in cubosome formulations than in 5FU alone. They could not detect a significant variation in the IC_50_ in HepG2 cells during the in vitro cytotoxicity investigation. These findings indicated that the cubosome formulation of 5FU did not affect the drug’s cytotoxicity. The non-significant result suggested that the medication alone caused the observed cell toxicity, not by the cubosome particle [116].

Albendazole is a potent inhibitor of numerous solid tumors. They are effective against hepatocellular cancer [117]. Albendazole’s low bioavailability, however, remains a concern in malignancies. Albendazole inhibits cancer via interacting with microtubules and inhibiting tubulin formation. Saber and colleagues came up with a cubosome formulation to look into the possibility of better bioavailability and the likely mechanism of albendazole’s anticancer action. They created albendazole-loaded cubosome dispersions utilizing GMO and P407 stabilizers. The findings indicated that the cubosome formulation of the drug resulted in a two-fold increase in bioavailability and more significant tumor regression in a rat model of cancer. Their investigation revealed that albendazole might prevent liver cancer by altering the ERK1/2-HIF-1-p300/CREB pathways via the cubosome formulation [99].

Natural products, such as extracts, isolated chemicals, and analogs, have been investigated as a possible medication or lead molecule in cancer treatment. Among these is gambogenic acid, a naturally occurring chemical derived from Gamboge, a herb used in traditional Chinese medicine. They demonstrated significant anticancer activity in preclinical tests through cell cycle arrest and cyclin D modulation. However, disadvantages such as poor solubility, short shelf life, irritation of blood vessels, and light sensitivity precluded it from clinical use. Luo and colleagues sought to synthesize gambogenic acid cubosomes using GMO and the stabilizer F127. They assessed its physiochemical characteristics and antitumor activity against SMMC-7721 human hepatocellular cancer cells. They successfully generated spherical or ellipsoidal monocellular cubosomes with exceptional cytotoxicity in SMMC-7721 cells. In vivo investigations have shown that cubosome formulations had a higher Cmax and AUC than the medication alone [100]. Another natural substance, Resveratrol, is a stilbenoid, a natural phenol that has shown a strong affinity for hepatocellular carcinoma cells. As with gambogenic acid, resveratrol has been associated with limited water solubility, low bioavailability, and photosensitivity. Abdel-Bar and colleagues overcame these challenges by preparing resveratrol cubosomes using a GMO and a P407 stabilizer. The cytotoxicity experiment revealed that the cubosome formulation was more cytotoxic in vitro to hepatic HepG2 cells. Additionally, they found improved drug uptake by cells. [35].

### 5.3. Cubosomes in Breast Cancer

Breast cancer is the leading cause of cancer in women globally. It is treatable in up to 80% of patients if found early and without metastases. It is difficult to treat cancer patients who have advanced to metastasis with current therapies. Breast cancer is classified as a heterogeneous illness at the molecular level due to epidermal growth factor receptor 2, hormone receptors, and many mutations [118]. Breast cancer treatment plans are determined by their molecular subtypes and include surgical intervention and chemotherapy. There are inadequate medication penetration, low bioavailability, and stability as with other solid tumors. Numerous attempts have been made to solve the problems related to breast cancer therapy by incorporating nanoparticles [119].

5- Fluorouracil is a commonly used medication in treating breast cancer, especially triple-negative breast cancer. As an antimetabolite of pyrimidine analog, it modulates several apoptotic pathways in breast cancer. It is quickly absorbed into systemic circulation through blood vessels, resulting in low drug concentration levels at the tumor site [120]. This will result in a decrease in efficacy as well as increased toxicity. Astolfi and colleagues created a bulk phase cubosome dispersion for 5-fluorouracil. To prepare the cubosomes, they employed phytantriol and Pluronic F127 stabilizer. In vitro cytotoxicity testing in the MDA-MB-231 cell line demonstrated that cubosomes containing 5-fluorouracil have higher cytotoxicity in the chosen cells than the medication alone [101].

Many naturally occurring chemicals have shown remarkable anti-breast cancer action. Thymoquinone is an active chemical derived from the plant *Nigella sativa* that effectively treats breast cancer. Their clinical applicability is hampered by properties such as low bioavailability and the lack of a measurement procedure in blood and tissues. Mehanna and colleagues generated thymoquinone-loaded cubosomes using an emulsification homogenization process. They compared the effects on estrogen-positive MCF-7 and estrogen-negative MDA-MB-231 cells to normal breast cells (MCF-10A). Containing a mean particle size of 98 nm, a prepared cubosome with GMO and pluronic F127 stabilizer demonstrated excellent entrapment effectiveness. They discovered a dose-dependent and time-dependent increase in apoptotic cells when treated with cubosome formulation against thymoquinone alone. Furthermore, drug accumulation in cells was better in cubosomes containing drugs [102].

### 5.4. Cubosomes in Lung Cancer

Lung cancer is becoming more common around the world. According to GLOBACAN, lung cancer became the most common disease and cancer-related mortality in 2019 when men and women were combined. With two major types: non–small-cell lung cancer (NSCLC) and small-cell lung cancer (SCLC), the most common cause of lung cancer is still smoking, although other variables such as asbestos and biomass burning air pollution are also linked to lung cancer. Despite several chemopreventive drugs being available for lung cancer, inconsistent outcomes make clinical recommendations challenging. Despite the availability of numerous modern medical techniques such as surgery, chemotherapy, and radiation therapy, treating lung cancer is getting more complicated as time passes. The main issue with chemotherapy in lung cancer is the lack of accuracy and the negative effects of therapeutic dosages. In this regard, nanotechnology plays a critical role in offering an appropriate delivery method [121,122].

Bedaquiline is a medicine that the FDA has licensed to treat TB, and it has shown great success in the inhibition of lung cancer. However, because of the drug’s limited water solubility, it has a significant difficulty in reaching the lungs. For this reason, Patil and colleagues created bedaquiline-loaded cubosomes that were specifically designed to target NSCLC. They made the inhalable cubosomes using GMO and the stabilizer Poloxamer 188. They could create a cubosome with a 51 percent encapsulation capacity and particle sizes of 150 nm. After nebulization, the cubosome showed excellent aerodynamic properties with a Mass median aerodynamic diameter 4.21 ± 0.53 µm. The cytotoxicity analysis revealed that the bedaquiline-loaded cubosomes exhibited considerable cytotoxicity in A549 cells through apoptosis. It is the first research to use cubosomes in inhalation treatment, and it is the most promising [103].

Lumefantrine, or benflumetol, is a well-known antimalarial drug. Numerous investigations indicate that this medicine may be employed as an anticancer agent in various solid tumors. It has a few drug delivery issues, including limited solubility in water and low bioavailability. Sethuraman and colleagues produced cubosomes filled with Lumefantrine calcium phosphate nanoparticles to investigate site-specific delivery in lung cancer. They created the cubosome by monolinolein, pyridinylmethyl linoleate, and Poloxamer 188 stabilizers. The cubosomes had shown encapsulating capacity of 78 % and a particle size of 259 nm. In A549 cells, the cubosomes formulation demonstrated significantly greater anticancer and anti-angiogenesis action than the medication alone [104].

### 5.5. Cubosomes in Cervical Cancer

Even though cervical cancer is the third most frequent disease in women, it is the most common cause of cancer in 40 low-income nations. It usually affects middle-aged women, and the major reason for this is the prevalence of the human papillomavirus (HPV). It is a condition that can be avoided, and getting the HPV vaccination at a young age may assist substantially with cervical cancer prevention. When a middle-aged woman is diagnosed with cervical cancer, treatment starts with surgical excision of early lesions, followed by chemotherapy and radiation. Cervical cancer therapy is currently costly, invasive, nonspecific, and unsatisfactory. As a result, Nanotechnology has been used to circumvent these problems, allowing for precision and target delivery with fewer adverse effects [123].

Doxorubicin is a commonly used anticancer medicine that is particularly effective against cervical cancer. However, free radical development of doxorubicin–iron complexes in the bloodstream increases the drug’s toxicity. Co-administration of antioxidant treatment is being utilized to reduce its adverse effects. A combination of external irradiation and doxorubicin has been used to minimize the therapeutic dosage of doxorubicin and its negative effects. External radiotherapy may be used to sensitize ionizing radiation to radionuclides associated with chemotherapeutic drugs. Cytryniak and colleagues established a dual-modality drug delivery method in Hela cervical cells in vitro, employing cubosomes for internal irradiation, paired with doxorubicin. The system is composed of GMO, a Pluronic F127 stabilizer, and 177Lu, a radionuclide with a low-energy beta (β^−^)-emitter. They discovered that cubosomes alone are non-toxic to Hela cells up to 54 µg/mL GMO. They detected a somewhat higher IC_50_ (15 MBq/mL) but statistically significant cytotoxicity at shorter time points, such as 24 h, with the cubosomes formulation [105].

Paclitaxel has been shown to be an effective chemotherapeutic agent for cervical cancer. The primary adjuvants utilized in the commercial formulation of paclitaxel for clinical use are polyethoxylated castor oil and dehydrated ethanol. Both of these contribute to the formulation’s homogeneity. However, this adjuvant combination is quite toxic. As a result, Aleandri and colleagues developed a cubosome formulation of paclitaxel to decrease toxicity and improve the site-of-action specificity. The cubosomes were generated utilizing GMO and the stabilizer PF108-B. The cubosome formulation was described, and its effectiveness was determined in Hela cells. The findings indicated that biotinylated cubosomes had more active functional biotin on the cell surface and exhibited more antitumor efficacy than paclitaxel alone. Additionally, the biotinylated cubosomes facilitated drug uptake at the cellular level. As a result, the formulation may be utilized in place of the traditional paclitaxel formulation to minimize adverse effects [106].

### 5.6. Cubosomes in Ovarian Cancer

Ovarian cancer is the fourth leading cause of mortality from cancer in women. It usually occurs in postmenopausal women, and the mortality rate is significant owing to the late presentation of the disease in clinics. Cancers are often treated surgically with hormone therapy, immunotherapy, chemotherapy, and radiation. However, the high likelihood of treatment resistance, adverse side effects, and relapse complicate care. Thus, novel tactics in pharmaceutical development are critical for minimizing adverse effects and ensuring effective medication delivery to the target location [124].

Icariin is an isolated phytoconstituent from the Chinese traditional medicinal herb *Herba epimedii*. Previous investigation has investigated its impact on ovarian cancer and its ability to trigger apoptosis through the PI3K/AKT and Raf1/ERK1/2 signaling cell-death pathways [125]. While it has a substantial anticancer effect in ovarian cancer, its low water solubility limits its bioavailability. Additionally, it has been shown that Icariin is metabolized into an inactive form by deglycosylation. As a result, Fahmy and colleagues improved Icariin-cubosomes and evaluated their efficiency in vitro in ovarian cells (SKOV-3 and Caov 3). Cubosomes were synthesized utilizing GMOs and a P407 stabilizer. The findings indicate that Icariin-cubosomes exhibit increased cytotoxicity in SKOV-3 and Caov 3 cells but not in normal EA.hy926 endothelial cells. The observed results might be a consequence of the chemicals’ increased solubility in the cubosome formulation [107].

Paclitaxel has been used to treat aggressive ovarian cancer similarly to how it is used in cervical cancer. However, as previously discussed, the difficulties connected with paclitaxel, such as adjuvant toxicity and significant side effects, remain the same. Zhai and colleagues developed cubosome formulations containing paclitaxel to enhance the effectiveness, while minimizing its adverse effects. The formulation comprised GMO and Pluronic F127 stabilizer, and was functionalized with EGFR fragments to improve tumor site targeting. They noticed a substantial drug loading capacity in the cubosome formulation. Additionally, the paclitaxel cubosomes demonstrated increased cytotoxicity in ovarian cells (HEY) and a 50% reduction in tumor burden in an animal xenograft model. The cubosome paclitaxel formulation outperformed paclitaxel alone in animal survival throughout the study. This demonstrates the formulation’s exceptional safety qualities [108].

### 5.7. Cubosomes in Skin Cancer

Skin cancer is a broad category that refers to various skin-related carcinomas, including basal cell carcinoma, cutaneous squamous cell carcinoma, and melanoma [126]. It is the most prevalent kind of cancer among Caucasians, and the most apparent causes are exposure to UV radiation and an aging population. Chemotherapy is the most successful treatment option for a variety of cancers. Still, when it comes to skin cancer—particularly melanoma, the most common kind of skin cancer—chemotherapy becomes exceedingly unsuccessful and unsatisfying. The main rationale for this is medication resistance caused by the disease’s unique traits. In skin cancer, drug resistance develops either due to acquired resistance during cytostatic drugs or as a result of inherent resistance. Thus, the hurdles in treatment strategies include overcoming resistance and increasing the quantity of drugs reaching tumor regions [127].

Cubosomes have been used to circumvent the difficulties associated with chemotherapy in skin cancer. Thus, Zhai and colleagues chose paclitaxel as the active ingredient in cubosome formulations, to evaluate human epidermal carcinoma A431 and an animal skin cancer xenograft model. The preparation was formulated using GMO and Pluronic F127, and its cytotoxicity was determined using A431 cells. In vitro cell assays revealed that the paclitaxel cubosome formulation was more tolerable than the medication alone. In the xenograft model, it was extremely evident that loaded paclitaxel accumulated preferentially at the tumor location. Additionally, when paclitaxel was loaded, the average tumor was decreased to half of its original size when compared to the medication alone [109]. Similarly, another natural substance, resveratrol, has been evaluated in cubosome formulations for the treatment of skin cancer. Resveratrol has previously shown anti-melanoma efficacy, although performance was determined to be suboptimal because of limited bioavailability. Kurangi and colleagues synthesized Resveratrol-loaded Cubosomal Gel from GMO and evaluated it in the skin layer of mice. The formulation improved skin permeability and deposition at the place of application in the mouse skin layer. The bioavailability investigation indicated that this compound has good potential for skin localization [110].

## 6. Cancer Theranostics and Cubosomes

**Figure 6:**
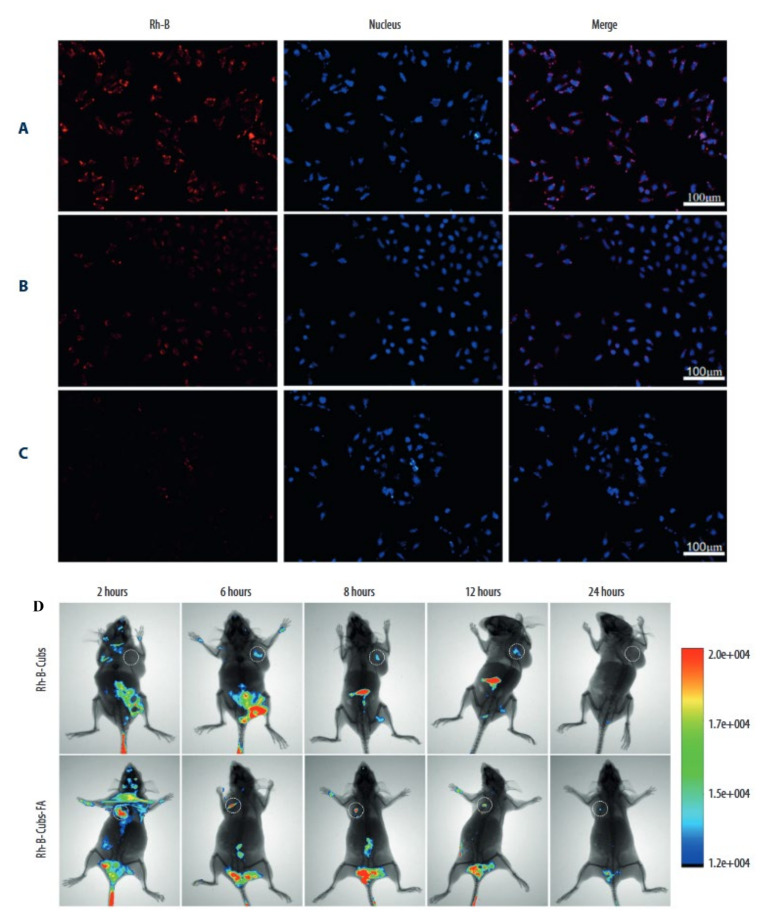
Rhodamine B (Rh-B) uptake by MCF-7 and in vivo tumor-targeting properties in mice bearing MCF-7 xenografts: (**A**) uptake of Rh-B; (**B**) uptake of Rh-B-Cubs; and (**C**) uptake of Rh-B-Cubs-FA at 4 h in MCF-7 cell lines; (**D**) whole-body and tumor fluorescence images (white circles indicate the inoculated tumor) in MCF-7 tumor-bearing mice after intravenous injection of Rh-B-Cubs and Rh-B-Cubs-FA. Source: Adapted from [129].

The word “theranostic” refers to the combination of therapy and diagnostics in treating any condition. Indeed, theranostic derives from the Greek words ‘thera’, which means therapeutic, and ‘nostic,’ which means diagnostic. Theranostic was developed to alleviate patient suffering and expedite therapy after diagnostics in cancer. Identifying biomarkers that are expressed in malignant cells but not in normal cells is critical in nanomaterial-based cancer theranostics. Additionally, the formulation’s nanomaterials must be safe, inert, and biocompatible in systemic settings [128]. A few efforts have been undertaken to realize cubosomes’ theranostic potential in cancer. Zhang and colleagues, for example, used RYLO and a stabilizer called Poloxamer 407 to create cubosomes. They used cisplatin and paclitaxel, and the cubosome was coated with poly-ε-lysine to avoid the immediate effects of the drugs, and to give long-term drug delivery and increased effectiveness. They used HepG2 cells to demonstrate that cubosome drug delivery might occur sustainably. The therapeutic potential of cubosomes was measured in cells by utilizing impedance measurement and fluorescent imaging [43]. In a different study, the surface modification of cubosomes was investigated to confer theranostic activity on cancer cubosome formulations, particularly those containing folates. Given the widespread availability of folate receptors in numerous tumor locations, it is believed that cubosomes conjugated with folate might enable selective targeting of cancer treatment sites. Thus, Tian and colleagues developed folate-modified cubosomes that contained etoposide stabilized with GMO and P407 and evaluated them in vitro on MCF-7 cells and animal models. They identified a considerable increase in drug accumulation when folate cubosomes were employed, which was shown using Rhodamine B (Rh-B)-loaded targeted cubosomes against non-targeted cubosomes (Rh-B-Cubs) and free Rhodamine B (Rh-B) (Figure 6). Meanwhile, in vivo tumor targeting properties in mice bearing MCF-7 xenografts showed that Rh-BCubs-FA is more successful at targeting tumor cells than Rh-B-Cubs (Figure 6). Thus, the produced cubosomes revealed potential Theranostic qualities when combined with imaging and therapeutic capabilities [129]. Park and colleagues demonstrated this receptor–ligand interaction with folate in another study using doxorubicin folate cubosomes. They discovered that functionalized cubosomes containing folate-delivered doxorubicin works more efficiently. Increased anticancer efficacy through apoptosis in an in vitro Hela cell culture was observed [130]. Additionally, Godlewska and colleagues observed a similar pattern of anticancer efficacy in folate-decorated monoolein cubosomes vs. folate-free cubosomes [131]. Collectively these studies indicate that there is a fast and augmented utilization of cubosomes in drug delivery and diagnostic application in cancer through theranostics intervention.

## 7. Concluding Remarks

Cubosomes are a bicontinuous cubic-phase colloidal dispersion in water that stabilize surfactants to generate a unique, nanoscale, structured system. They generally have a diameter of 10 to 300 nm and are used to transport a variety of chemical substances in both living and non-living things. They have many characteristics such as bio-adhesives, improved dermal penetration, simplicity of formulation, more drug loading capacity, greater stability at any dilution level, higher resistance to breaking, and protecting enzyme attack-prone pharmaceuticals inside the cubic phase. Moreover, they are low-cost, physiologically friendly, and non-hazardous. However, the generation of increased viscosity during large-scale manufacturing and a few concerns with hydrophilic drug retention remain its key downsides. As a drug delivery system, cubosomes have been demonstrated to be effective in a variety of dosage forms, including oral, topical, ocular, and parenteral administration. The key benefits of cubosomes are their ability to accommodate poorly water-soluble medicines and target specific sites in the body. In particular, in the field of cancer, several advanced studies are being conducted to combine anticancer medications into cubosome formulations as drug carriers, with the expectation of a significant improvement in cancer treatment. Using the cubosome anticancer medication delivery system, researchers have shown that the risk of adverse effects to the patient is very minimal. This is in line with the other research that has been conducted so far. As a result, it lessens the unpleasant sensations that cancer patients endure throughout their therapies. The inclusion of theranostics has significantly increased the therapeutic value of anticancer drugs that mainly target the tumor location, while also providing the additional advantage of accommodating diagnostic applications at the same time. In the future, cubosome-mediated targeted nanoparticle cancer-drug carriers have the potential to revolutionize cancer therapy by improving the quality of life of cancer patients. However, many improvements must be made in the technological aspects of cubosomes before they can be used in clinical practice.

## Figures and Tables

**Table 1:** Applications of cubosomes for anticancer drug delivery.

Sl No.	Cancer/ Cells Type	Chemicals/Drugs	Polymer Used	Stabilizer	Findings	Ref
1	Colorectal/HCT-116	Cisplatin	GMO	Pluronic F127	Cisplatin-loaded nano-cubosomes decreased the cell viability of HCT 116 and augmentation of their cytotoxicity in the presence of metformin.	[94]
2	Colorectal/HCT-116 and Caco-2	Metformin	GMO	Pluronic F127	The cubosomes formulation significantly lowered the IC_50_ concentration at which viable cells were destroyed compared to metformin alone.	[95]
3	Colorectal/HT-29	Cornelian cherry	GMO	Poloxamer^®^ 407	After 24 and 48 hours of incubation, Cornus mas extract cubosome improved IC_50_ value 1.33 and 1.47 times higher than free Cornus mas extract. The cubosome formulation stopped G1 phase cell growth and produced apoptosis in the cancer cell line HT-29.	[96]
4	Colorectal/Caco-2	20(S)- protopanaxadiol	GMO	Poloxamer^®^ 407	The PPD-cubosome showed higher bioavailability, and better release was which is likely owing to greater absorption by the cubic nanoparticles.	[97]
5	Hepatic/HepG2	5-Fluorouracil	GMO	Poloxamer^®^ 407	5-FU-loaded cubosomes performed well in vitro cell culture. The cubosomes formulation also boosted bio distribution concentration of 5-FU in the liver compared to the 5-FU solution alone in the rat.	[98]
6	Hepatic/rat model	Albendazole	GMO	Poloxamer^®^ 407	The cubosome formulation of the drug resulted in a two-fold increase in bioavailability and greater tumor regression in a rat model of cancer.	[99]
7	Hepatic/SMMC-7721	Gambogenic acid	GMO	Poloxamer^®^ 407	The prepared spherical or ellipsoidal monocellular cubosomes showed remarkable cytotoxicity in the SMMC-7721 cells.	[100]
8	Hepatic/HepG2	Resveratrol	GMO	Poloxamer^®^ 407	The cubosome formulation had higher cytotoxicity against hepatic HepG2 cells in vitro, and superior cell internalization of drugs was observed.	[35]
9	Breast/MDA-MB-231	5- Fluorouracil	Phytantriol	Pluronic F127	In vitro cytotoxicity testing in the MDA-MB-231 cell line demonstrated that cubosomes containing 5-fluorouracil exhibit more cytotoxicity in the chosen cells than the medication alone.	[101]
10	Breast/MDA-MB-231/MCF-7	Thymoquinone	GMO	Poloxamer^®^ 407	A dose and time-dependent increase in apoptotic cells was observed when treated with Thymoquinone-cubosome formulation against Thymoquinone alone.	[102]
11	Lung/A549	Bedaquiline	GMO	Poloxamer 188	The findings revealed that the cubosome formulation containing the medication exhibited considerable cytotoxicity in A549 cells, in addition to inducing apoptotic cell death, and had anti-invasive properties.	[103]
12	Lung/A549	Lumefantrine	GMO	Poloxamer	In A549 cells, the cubosomes formulation demonstrated significantly greater anticancer and anti-angiogenesis action than the medication alone.	[104]
13	Cervical/Hela	Doxorubicin	GMO	Pluronic F127	There was somewhat higher IC_50_ (15 MBq/mL) but statistically significant cytotoxicity at shorter time points, such as 24 h, with the cubosomes formulation.	[105]
14	Cervical/Hela	Paclitaxel	GMO	PF108-B	The biotinylated cubosome facilitated drug uptake at the cellular level.	[106]
15	Ovary/SKOV-3 and Caov 3	Icariin	GMO	Poloxamer^®^ 407	The findings indicate that Icariin-cubosomes exhibit considerably increased cytotoxicity in both SKOV-3 and Caov 3 cells, but not in normal EA.hy926 endothelial cells.	[107]
16	Ovary/HEY	Paclitaxel	GMO	Pluronic F127	The paclitaxel cubosomes demonstrated increased cytotoxicity in ovarian cells (HEY) and a 50% reduction in tumor burden in an animal xenograft model with more safety feature.	[108]
17	Skin/A431 cells	Paclitaxel	GMO	Pluronic F127	Loaded paclitaxel accumulated preferentially at the tumor location. Additionally, when paclitaxel was loaded, the average tumor size was decreased to half of its original size when compared to the medication alone.	[109]
18	Skin/mice	Resveratrol	GMO	Pluronic F127	The formulation improved skin permeability and deposition at the place of application in the mouse skin layer.	[110]
